# Moral Injury, Depression and Anxiety among Israeli Health and Social Care Workers During the COVID-19 Pandemic: The Moderating Role of Thwarted Belongingness

**DOI:** 10.1192/j.eurpsy.2022.1361

**Published:** 2022-09-01

**Authors:** G. Zerach, Y. Levi-Belz

**Affiliations:** 1 Ariel University, Psychology, Ariel, Israel; 2 Ruppin Academic Center, Behavioral Sciences, hadera, Israel; 3 Ruppin Academic Center, The Lior Tsfaty Center For Suicide And Mental Pain Studies, Emek Hefer, Israel

**Keywords:** Moral Injury, Anxiety, Covid-19, Depression

## Abstract

**Introduction:**

The COVID-19 pandemic can affect the mental health of health and social care workers (HSCWs) who are frontline workers in this continuous crisis. Following exposure to potentially morally injurious events (PMIEs) that undermine deeply held moral beliefs and expectations, HSCWs might experience moral injury (MI) and deleterious psychiatric consequences such as depression and anxiety symptoms.

**Objectives:**

To examine associations between exposure to PMIEs, MI symptoms, depression, and anxiety symptoms. We also aim to assess the moderating role of thwarted belongingness in these associations.

**Methods:**

A sample of 243 Israeli HSCWs completed online validated self-report questionnaires in a cross-sectional designed survey in February and March 2021.

**Results:**

About one-third (33.6%) of the sample met the criteria for major depressive disorder, 21.5% met the criteria for generalized anxiety disorder, and 19.1 % reported comorbidity of depression and anxiety. A moderated-mediation model shows that high thwarted belongingness intensified the relations between exposure to PMIEs and MI symptoms, and between MI symptoms and depression and anxiety symptoms. Importantly, the indirect effect of exposure to PMIEs on both depression and anxiety symptoms via MI symptoms existed only among those with high levels of thwarted belongingness.

**Conclusions:**

The study’s findings highlight the mental burden of HSCWs during the COVID-19 pandemic and the contribution of MI to possible mental health consequences. Clinicians should be aware of the importance of high thwarted belongingness in depression and anxiety sequelae of exposure to PMIEs among HSCWs.

**Disclosure:**

No significant relationships.

